# Pretreatment Lymphocyte Monocyte Ratio Predicts Long-Term Outcomes in Patients with Digestive System Tumor: A Meta-Analysis

**DOI:** 10.1155/2016/9801063

**Published:** 2016-08-09

**Authors:** Jingwen Zhang, Lishan Chen, Rui Zhou, Huiying Sun, Yulin Liao, Wangjun Liao

**Affiliations:** ^1^Department of Oncology, Nanfang Hospital, Southern Medical University, Guangzhou 510515, China; ^2^Huiqiao Medical Center, Nanfang Hospital, Southern Medical University, Guangzhou 510515, China; ^3^Department of Cardiology, Nanfang Hospital, Southern Medical University, Guangzhou 510515, China

## Abstract

*Purpose.* The prognostic value of pretreatment lymphocyte monocyte ratio (LMR) in digestive system cancer patients remains controversial. The aim of this study was to quantify the prognostic impact of this biomarker and assess its consistency in digestive system tumors.* Methods.* We searched “PubMed,” “Embase,” and “CBM” for published eligible studies before June 2016 and conducted a meta-analysis to estimate the pooled hazard ratios (HRs) for disease recurrence and mortality focusing on LMR. Subgroup analyses, meta-regression, and sensitivity analyses were also performed.* Results.* A total of 22 cohort studies enrolling 12829 patients with digestive system cancer were included. The summary results showed that lower LMR was significantly associated with worse overall survival (OS), cancer-specific survival (CSS), and tumor disease or recurrence-free survival (DFS/RFS) in analyses using the studies reporting HRs either by the univariate analyses (HR = 1.32, HR = 1.35, and HR = 1.26 for OS, CSS, and DFS/RFS, resp.) or by multivariate analyses (HR = 1.21, HR = 1.18, and HR = 1.26 for OS, CSS, and DFS/RFS, resp.).* Conclusion.* Our results support the fact that decreased LMR indicates worse prognosis in multiple digestive system tumors.

## 1. Introduction

There is increasing evidence showing that the tumor microenvironment (TME) and TME-related pathways weigh a lot in tumor growth, invasion, and metastasis [1, 2]. Indeed, the crosstalk between tumor cells and their ambient TME determines the outcome of these biological processes [3]. Recently, the close relationship between systemic inflammatory response, an important component of TME, and cancer development has been gradually taken into concern [4]. Inflammation regulating factors and effector cells are shown to take part in various carcinogenetic events [5]. Meanwhile, some peripheral blood inflammatory parameters are found to have prognostic prediction values in cancer patients [6, 7]. Obviously, compared with immunohistochemical markers that largely depend on the resection or biopsy of tumor samples, obtaining peripheral blood samples is convenient, less invasive, and easier for dynamic evaluation. Among these markers, the pretreatment lymphocyte monocyte ratio (LMR) in particular has gained notable interest recently. There has been only two meta-analyses which revealed that an elevation of LMR was likely to indicate a better prognosis in various solid tumors [8, 9]. However, the consistency and magnitude of the prognostic impact of LMR, especially in digestive system tumors, still lack systematic analyses to confirm. Accordingly, we conducted a systematic review and meta-analysis in the hope of identifying the clinical value of pretreatment LMR elevation in predicting long-term outcomes for digestive system tumors.

## 2. Materials and Methods

### 2.1. Literature Search and Study Selection

Systematic computerized search of PubMed, Embase, and Chinese Biomedical Literature Database (CBM) was conducted in June 2016. The following keywords were used in various forms and combinations for “Title/Abstract” based search: “cancer”, “tumor”, “carcinoma”, “neoplasm”, “adenocarcinoma”, “malignant”, “oncology”, “lymphocyte monocyte ratio”, and “monocyte lymphocyte ratio”. Additionally, “neoplasm” was also used in “Medical Subject Headings” based search. An example of initial search strategy using recognized search terms was provided in Supplementary Material (see Supplementary Material available online at http://dx.doi.org/10.1155/2016/9801063). References listed within selected studies were also searched for potentially eligible studies. Corresponding authors were contacted for further information if necessary. Study was conducted according to the Preferred Reporting Items for Systematic Reviews and Meta-Analyses (PRISMA) statement [10].

The abstracts of all candidate articles were read by two independent reviewers (Zhang and Chen). Articles that could not be categorized based on title and abstract alone were retrieved for full-text review. These articles were independently read and checked for inclusion criteria. Any disagreements were resolved through consensus with a third reviewer (Zhou).

### 2.2. Study Inclusion/Exclusion Criteria

Inclusion criteria for primary studies were as follows: (a) patients were diagnosed as digestive system tumors; (b) more than 10 patients were involved; (c) the correlation between pretreatment LMR value and survival information was investigated; (d) the study was original; (e) the study was published as a full-text paper in either English or Chinese.

Studies were excluded based on the following criteria: (a) letters, reviews, case reports, editorials, expert opinion, or laboratory studies; (b) studies that had duplicate data or repeat analysis; (c) cancer treatments prior to obtaining peripheral blood samples; (d) lacking of key information for further analysis, such as survival information; (e) nonhuman research.

### 2.3. Quality Assessment of Primary Studies

The quality assessment of the included studies was performed by the three primary reviewers (Zhang, Chen, and Zhou) independently. The quality of all the 22 acceptable studies elaborating LMR and survival information was evaluated using the Newcastle-Ottawa Quality Assessment Scale (NOS) for cohort studies (Table S1). The modified NOS (http://www.ohri.ca/programs/clinical_epidemiology/oxford.asp) of non-RCTs addressed the following three items: patient selection, comparability of groups, and outcome assessment. Stars were given to high-quality elements and the total was used for study quality comparison in a quantitative manner. We considered a study awarded seven or more stars as a high-quality study [11].

### 2.4. Data Extraction

Data were independently extracted from the eligible articles by two reviewers (Zhang and Chen). The following data were collected from each of the studies: study characteristics (author name, year of publication, and number of patients), patient characteristics (age, gender, and country), tumor site, anatomic structure type, distant metastatic status, treatment strategy, the lower limit of elevated LMR, the portion of patients whose LMR values were higher than cut-off, and survival information (follow-up months, whether multivariate analysis (MVA) was conducted, and the method of getting univariate analysis (UVA) hazard ratio (HR)).

If HRs and their confidence intervals (CIs) were not directly reported but the information of the number of patients with high and low LMR levels together with the number of observed deaths or disease recurrences was available, mathematical HR was estimated using the established method [12]. In the case when sufficient data were not directly available but a Kaplan-Meier curve was provided, we estimated HR through the extracted data from the Kaplan-Meier curve using the same method [12]. If none of the above information was reported, the study then was excluded.

### 2.5. Descriptive Statistics and Meta-Analysis

We performed statistical analysis using RevMan software version 5.1 (Cochrane Collaboration, Oxford, UK) and the META module of STATA version 12.0 (Stata Corporation, College Station, TX). Considering the association between LMR and clinicopathological variables and that such relativity might also be one of the reasons why LMR level influences patient prognosis, we conducted our meta-analysis using HRs from UVA and MVA separately.

For survival analysis, the pooled HRs were calculated. An observed HR > 1 indicated worse outcome for the study group relative to the reference group. Heterogeneity among studies was examined using *I* ^2^ [13]. Substantial heterogeneity was defined as an *I* ^2^ > 50%. A fixed-effects model was applied when *I* ^2^ < 50%; otherwise a random-effects model was used. The prespecified subgroup analyses were performed using the following variables: anatomic structure, cancer type, disease stage, patient ethnicity, cut-off value used, and treatment strategy. Meta-regression analyses were used to explore the potential heterogeneity contributors [14, 15]. In order to avoid data dredging, we firstly conducted meta-regression focusing on one single covariate at a time to figure out significant covariates. If there was more than one significant covariate, we then adjust them in corresponding models simultaneously to further identify independent covariates. Sensitivity analyses were also conducted by changing the effect models or estimating the average HR after sequential omission of each individual study. Publication bias was evaluated by Egger's test [16]. When the publication bias was observed, the trim and fill method was used to test the stability of the results [17]. Statistical significance was reached when *P* values < 0.05.

## 3. Results

### 3.1. Literature Search and Quality Assessment

**Figure 1:**
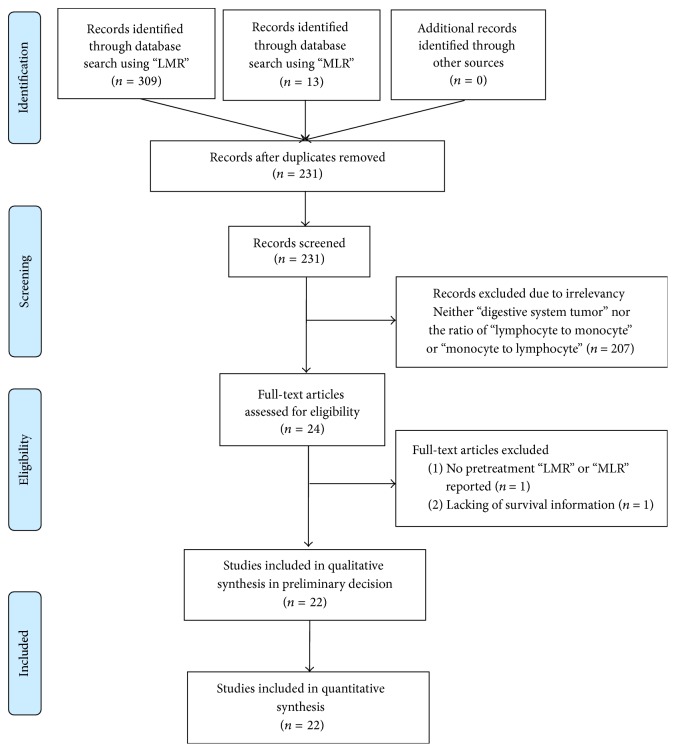
Selection of studies included in the analysis. LMR: lymphocyte to monocyte ratio.

The titles and abstracts of 322 primary studies were identified for initial review using searching strategies as described and no additional records were identified through the references listed within selected studies. A total of 22 studies [18–39] were finally included for systematic review following the PRISMA statement (Figure 1). The selection and quality assessment was performed on all 22 studies. The total stars of each of the studies were all more than seven stars, suggesting acceptable overall quality of the included studies. The results of selection and quality assessment were listed in Table S2.

### 3.2. Main Study Characteristics

A total of 12829 patients were included for the meta-analysis. Characteristics of included studies were shown in Table 1 and Table S3. In these studies, the lower limit of elevated LMR ranged from 2.3 to 4.95. The UVA HRs and their 95% CIs of included studies were collected, and ten of them from six studies were estimated using Kaplan-Meier survival curves, while others were directly reported. All studies we included conducted multivariable analysis except the study of Neal et al. [24].

### 3.3. Meta-Analysis of HRs from Univariate Analysis

All studies were included in the meta-analyses for survival based on UVA HRs. Eighteen studies reported overall survival (OS), six studies reported cancer-specific survival (CSS), and thirteen studies reported disease or recurrence-free survival (DFS/RFS). The main results were listed in Table 2.

**Figure 2:**
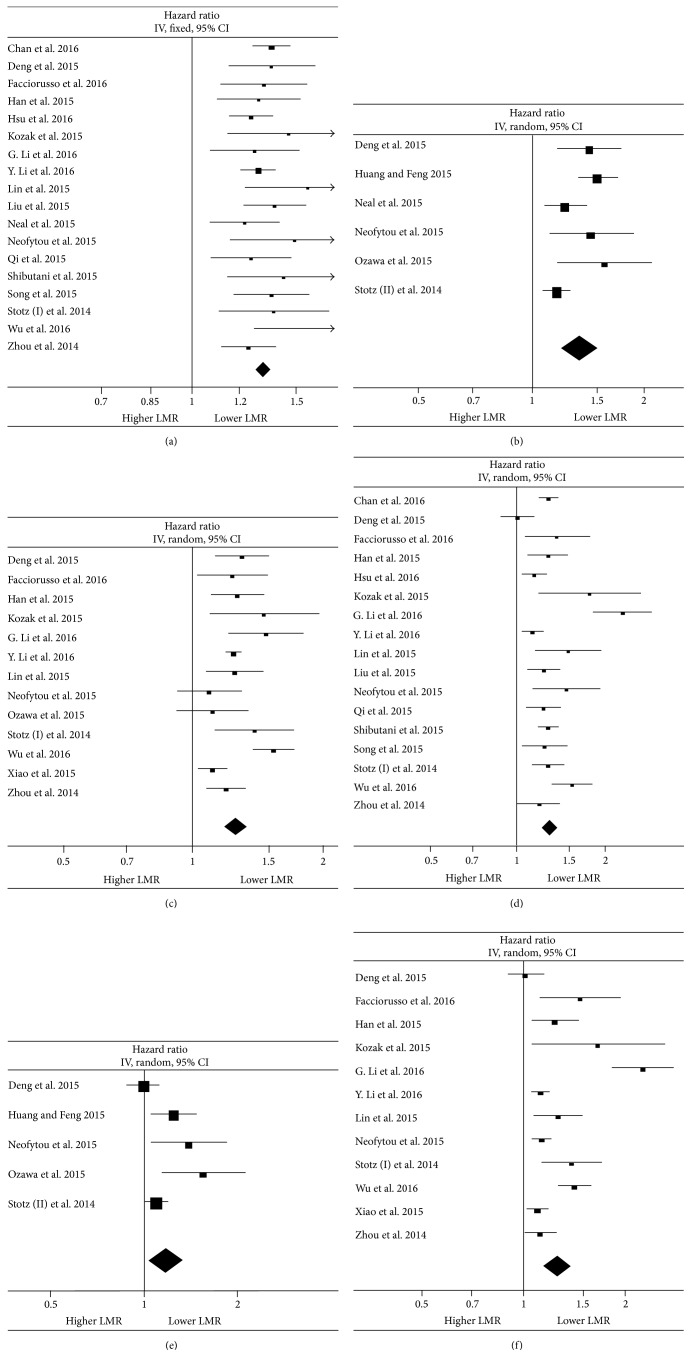
Main forest plots of meta-analysis. Forest plots of the association between lymphocyte to monocyte ratio and overall survival (OS), cancer-specific survival (CSS), and disease or recurrence-free survival (DFS/RFS). Hazard ratios (HRs) for each study are represented by the squares. The size of the data markers (squares) corresponds to the weight of the study in the meta-analysis, and the horizontal line crossing the square represents the 95% confidence interval (CI). All statistical tests were two-sided. (a) Meta-analysis of patients OS from univariate analysis (UVA); (b) meta-analysis of patients CSS from UVA; (c) meta-analysis of patients DFS/RFS from UVA; (d) meta-analysis of patients OS from multivariate analysis (MVA); (e) meta-analysis of patients CSS from MVA; (f) meta-analysis of patients DFS/RFS from MVA.

Through meta-analyses, we found that decreased LMR indicated not only a higher risk for patients overall mortality (HR 1.32, 95% CI 1.28–1.36; *I* ^2^ 0, Figure 2(a)), but also cancer-specific death (HR 1.35, 95% CI 1.20–1.50; *I* ^2^ 65%, Figure 2(b)) and tumor recurrence (HR 1.26, 95% CI 1.18–1.33; *I* ^2^ 63%, Figure 2(c)). Then subgroup analyses (Table 2) revealed that the significantly improved prognostic effect of lower pretreatment LMR for OS could be observed in all tumor sites (the largest effect size was observed in the patients with liver cancer), in cancers of anatomic structure of gastrointestinal tract or nongastrointestinal tract, in Asian or non-Asian patients, in non-metastasis, metastasis, or mixed tumor stages, and in patients receiving surgery or not. A separate analysis performed on cut-off values also identified lower LMR value as an unfavorable factor for improved prognosis in subgroups of the data applying “<3.0” or “≥3.0”. Concerning subgroup analyses of CSS and DFS (Table 2), the significant survival benefit was also observed in all subgroups regarding tumor site, anatomic structure, tumor stage, ethnicity, and cut-off value.

### 3.4. Meta-Analysis of HRs from Multivariate Analysis

MVA were performed in 21 studies. In meta-analysis of MVA HRs (Table 3), LMR less than the cut-off was associated with HRs for OS of 1.29 (95% CI 1.21–1.38; *I* ^2^ 73%, Figure 2(d)), for CSS of 1.18 (95% CI 1.04–1.34; *I* ^2^ 65%, Figure 2(e)), and for DFS/RFS of 1.26 (95% CI 1.16–1.38; *I* ^2^ 83%, Figure 2(f)). In subgroup analyses for OS, the decrease of OS in patients with lower LMR reached significance in all subgroups except in patients with pancreatic cancer. In terms of subgroup analyses for CSS, however, the LMR did not appear to be an independent prognostic factor in patients with mixed stage, in both subgroups according to patient ethnicity and in the LMR cut-off value ≥3.0 group. As for analyses of DFS/RFS, the independent positive prognostic effect of a low LMR on DFS/RFS was not seen in subgroups of gastric cancer patients. Meta-regression analyses showed that the tumor site and the anatomic structure type were observed to be heterogeneity contributors for both OS and DFS/RFS through meta-regressions based on single variables. When combining both tumor site and the anatomic structure type simultaneously, only the anatomic structure type maintained its significance for OS (*P* = 0.016), while the anatomic structure type (*P* = 0.015) and tumor site of liver (*P* = 0.018) still contributed to heterogeneity of DFS/RFS significantly independently. None of other variables were found to be potential sources of heterogeneity (Table 4).

### 3.5. Sensitivity and Publication Bias Analysis

We then used sensitivity analyses to evaluate the stability of the results of analysis considering all the included studies. No statistically significant change of the pooled estimated HRs for OS, CSS, and DFS/RFS was found when using the different effect models in meta-analysis of HRs from both UVA and MVA. In the leave-one-out sensitivity analyses, the result patterns were not obviously impacted by any single study for all analyses (data not shown). This indicated that our pooled results were stable. Also, we assessed for publication bias of main results by the Egger test (Tables 2 and 3). No evidence of significant publication bias was observed except meta-analysis of MVA OS and MVA DFS. So, we further used the trim and fill method to validate the reliability of corresponding results. The pooled analysis incorporating the hypothetical studies continued to show statistically significant associations between poorer MVA OS (*P* < 0.001) and MVA DFS (*P* < 0.001) with lower LMR. In summary, the results of sensitivity analyses and publication bias analyses supported the credibility of most of the evidence in this meta-analysis.

## 4. Discussion

Following the well-established and accepted concept that the pathogenesis of cancer is considered as an inflammation-driven malignancy [4], our current study for the first time comprehensively validated the clinical impact of pretreatment LMR in patients with digestive system tumor based on a large pool of clinical studies incorporating 12829 patients. We found a consistent harmful effect of decreased LMR on patient survival, including OS, CSS, and DFS/RFS in UVA pooled results. Such significant prognostic impact could be found among various disease subgroups, in organs with anatomic structure of gastrointestinal tract or nongastrointestinal tract, in Asian or non-Asian patients, regardless of metastatic stage and cut-off value, and in patients receiving surgery or not. With regard to the correlation between LMR and survival in MVA pooled results, similar negative effect of lower LMR was observed in total results of OS, CSS, and DFS/RFS. However, although subgroup analyses showed same trend of survival impact as total results, some results failed to reach significance. Moreover, substantial heterogeneity was observed in most results. This might be attributed to the fact that although HRs derived from MVA were results adjusted possible confounders at each study level, this still had an important limitation that was unavoidable: the different studies adjusted their MVA with different factors. This could impact the calculated multivariate HRs significantly and thus increase the risk of bias and heterogeneity. Therefore, caution must be exercised when interpreting the results. Meanwhile, whether LMR could act as an independent risk factor in digestive tumor still needs more studies to confirm.

However, the specific mechanism behind the association of higher LMR and favorable outcome of digestive system cancer patients still remains unclear. The unbalance between protumor and anticancer inflammatory status of hosts probably was the main reason [40, 41]. In TME, the lymphocyte was usually regarded as one of the most crucial components of the host's cellular immunity [42] and the cellular basis of immunosurveillance and immunoediting against nascent tumor cells [43, 44] through induction of tumor cell apoptosis [45]. Hence, a low lymphocyte count might be responsible for a weak, insufficient immunologic reaction to tumor and thereby a worsened clinical outcome. On the contrary, the role of macrophages/monocytes in cancer development and progression remains controversial. Previous data showed a protective effect of tissue-specific macrophages cells [46, 47], such as Kupffer cells, which could eliminate circulating tumor cells. However, following the increasing focus on tumor-infiltrating macrophages (TIMs) derived from circulating monocytes, researchers found that, unlike common macrophages, TIMs enhanced tumor progression [48, 49]. The excessive production of TIMs can stimulate the growth of tumor cells, enhance neoangiogenesis, and thereby promote tumor cell migration and metastasis [50, 51]. TIMs can also produce enzymes and inhibitors that regulate the digestion of the extracellular matrix and hence further favoring tumor invasion [52]. Moreover, these TIM-released soluble factors could also suppress the antitumor immune responses by making T cell subsets lack cytotoxic function [53]. The circulating level of monocytes can reflect the formation or presence of TIMs, which explained why an elevated monocyte count confers a negative prognosis in patients with digestive system tumors.

The association of clinicopathological factors and LMR level was also reviewed in some studies retrieved in our analysis. Firstly, in terms of T category, Chan et al. [34] Huang and Feng [20], Hsu et al. [36], Lin et al. [22], and Li et al. [38] reported that lower LMR value was significantly correlated with an increased likelihood of higher degree of tumor infiltration (or larger tumor size) in esophageal cancer, gastric cancer, colorectal cancer, and liver cancer, respectively. Then, when considering N category, lower LMR was also significantly related to a higher risk of positive status of lymph nodes metastasis in esophageal cancer [20] and gastric cancer [36]. Moreover, in the study of Kozak et al. [21] about colorectal cancer, patients with lower LMR had significantly higher rates of worse stage, which was also consistent with the results of Hsu et al. [36] in gastric cancer and Li et al. [37] and Stotz et al. [31] in pancreatic cancer. These findings suggested that LMR could be a predictor of the clinicopathological features in some digestive system tumors.

This meta-analysis had several limitations that must be taken into account in the interpretation. Firstly, only summarized data rather than individual patient data could be used. Secondly, this analysis was constrained to studies published in English language only, and most of the literatures we brought into our review were from Asian countries (especially from China) and were published in 2015. Although the “trim and fill” method has validated the reliability of the pooled results with publication bias, it still has to be noted that evaluation of publication bias could not be done in a robust manner with such few data points, and the statistical power of Egger's test to suspect publication bias was also kind of limited here. Thirdly, the cut-off values used by individual study varied from each other in the included studies, and the criteria method of selecting LMR cut-offs remained unclear. Last but not least, all included studies were retrospective single-center studies. Therefore it would be a little hard to control all kinds of factors that might affect patient survival and the level of inflammatory markers between two groups. Finally, obvious heterogeneity was observed for some of our analyses. Therefore, the random-effects model was used. Despite having tried several kinds of methods to figure out all sources of heterogeneity, the presence of heterogeneity might also result from many other factors, including age distribution, tumor size, and factors by which studies adjusted their multivariate analysis. In fact, since clinical and methodological diversity always occur in meta-analyses, statistical heterogeneity was inevitable [13]. Much more detailed data is needed to assess the heterogeneity in the future meta-regression. Of note, the results of meta-regressions in our study were only hypothesis-generating rather than confirmatory, since the number of studies included in the analysis was kind of limited, and the possible influential factors found by exploratory meta-regression may be subject to the false-positive conclusions because the false-positive rates could not be controlled completely [14].

## 5. Conclusion

In conclusion, this systematic review demonstrated the associations between lower LMR and poorer clinical outcomes in patients with digestive system tumors. LMR could be a convenient, easy-to-measure indicator for patients with a great clinical biological value in our future clinical practice.

## Supplementary Material

Example of search criteria in “PubMed”: The following keywords were used in various forms and combinations for “Title/Abstract” based search: “cancer”, “tumor”, “carcinoma”, “neoplasm”, “adenocarcinoma”, “malignant”, “oncology”, “lymphocyte monocyte ratio”, and “monocyte lymphocyte ratio”. Additionally, “neoplasm” was also used in “Medical Subject Headings” based search.
Table S1：This scale is an eight-item instrument that allows for assessment of patient population and selection, study comparability, follow-up, and outcome of interest. Interpretation of the scale is performed by awarding stars, for high-quality elements. Stars are then added up and use to compare study quality in a quantitative manner. A study can be awarded a maximum of one star for each numbered item within the Selection and Outcome categories. A maximum of two stars can be given for Comparability. Quoted phrases are provided in the scale to allow for adjustment to particular studies.
Table S2：The scale includes eight items in total with four items in selection category, one item in comparability category, and three items in outcome category. Stars were given to high-quality elements. Having seven or more stars is considered good quality.
Table S3: Summary table of studies included in meta-analysis. All studies were conducted between 2013-2016. Country described where the study conducted. Tumor site included colorectal, stomach, esophageal, and liver and pancreas. Treatment described whether the patients received surgical or not. Tumor stage was most often described according to distant metastasis status. For this table, studies were grouped as non-metastatic stage (NMS), metastatic stage (MS) or mixed stage. Clinical outcomes include overall survival (OS), cancer-specific survival (CSS), relapse-free survival (RFS), and disease-free survival (DFS).

## Figures and Tables

**Table 1:** Main characteristics of eligible studies.

Characteristics	Number of studies	Number of patients
*All eligible studies*	22	12829
*Year of publication*		
2014	3	1272
2015	13	2951
2016	6	8606
*Endpoint used*		
OS	18	11610
CSS	6	1770
DFS/RFS	13	8338
*Method to get UVA HR*		
Reported	16	10853
Estimated	6	1976
*Multivariate analysis*		
Performed	21	12527
Unperformed	1	302
*Anatomic structure*		
Gastrointestinal tract	17	11340
Nongastrointestinal tract	5	1489
*Tumor site*		
Esophagus	3	892
Stomach	3	1741
Colorectal	11	8707
Liver	2	660
Pancreas	3	829
*Stage*		
NMS	11	9412
Mixed	3	1789
MS	8	1628
*Ethnicity*		
Asian	16	11285
Non-Asian	6	1544
*Cut-off value*		
<3.0	10	9272
≥3.0	12	3557
*Treatment*		
With surgery	18	12210
No surgery	4	619

OS: overall survival; CSS: cancer-specific survival; DFS: disease-free survival; RFS: recurrence-free survival; UVA: univariate analysis; HR: hazard ratio; NMS: nonmetastatic stage; MS: metastatic stage.

**Table 2:** Meta-analysis of HRs from univariate survival analysis.

Outcome	Groups	Number of studies	Model	HR (95% CI)	*I* ^2^ (%)	Egger test
OS	*All study*	18	Fixed	1.32 [1.28–1.36]	0	0.156
	*Tumor site*					
	Esophagus	2	Fixed	1.35 [1.22–1.49]	0	
	Stomach	3	Fixed	1.27 [1.19–1.35]	0	
	Colorectal	9	Fixed	1.33 [1.28–1.39]	0	
	Liver	2	Fixed	1.67 [1.35–2.04]	0	
	Pancreas	2	Fixed	1.27 [1.12–1.43]	0	
	*Anatomic structure*					
	GT	14	Fixed	1.32 [1.28–1.37]	0	
	Non-GT	4	Fixed	1.35 [1.22–1.49]	44	
	*Stage*					
	NMS	9	Fixed	1.33 [1.28–1.39]	0	
	Mixed	3	Fixed	1.28 [1.19–1.37]	0	
	MS	6	Fixed	1.35 [1.25–1.45]	10	
	*Ethnicity*					
	Asian	13	Fixed	1.32 [1.28–1.37]	0	
	Non-Asian	5	Fixed	1.33 [1.22–1.45]	0	
	*Cut-off value*					
	<3.0	8	Fixed	1.32 [1.27–1.39]	0	
	≥3.0	10	Fixed	1.32 [1.25–1.39]	4	
	*Treatment*					
	With surgery	9	Fixed	1.32 [1.28–1.37]	0	
	No surgery	3	Fixed	1.33 [1.22–1.45]	0	

CSS	*All study*	6	Random	1.35 [1.20–1.50]	65	0.52
	*Tumor site*					
	Colorectal	3	Fixed	1.32 [1.18–1.47]	29	
	*Anatomic structure*					
	GT	5	Fixed	1.39 [1.28–1.52]	24	
	*Stage*					
	Mixed	2	Random	1.27 [1.04–1.54]	70	
	MS	3	Fixed	1.32 [1.18–1.47]	29	
	*Ethnicity*					
	Asian	3	Fixed	1.49 [1.35–1.64]	0	
	Non-Asian	3	Fixed	1.21 [1.12–1.28]	12	
	*Cut-off value*					
	<3.0	3	Random	1.28 [1.11–1.49]	80	
	≥3.0	3	Fixed	1.47 [1.27–1.69]	0	

DFS/RFS	*All study*	13	Random	1.26 [1.18–1.33]	63	0.717
	*Tumor site*					
	Stomach	2	Fixed	1.22 [1.12–1.33]	0	
	Colorectal	7	Fixed	1.19 [1.11–1.28]	46	
	Liver	2	Random	1.39 [1.14–1.69]	78	
	*Anatomic structure*					
	GT	10	Fixed	1.20 [1.18–1.25]	29	
	Non-GT	3	Random	1.41 [1.23–1.61]	58	
	*Stage*					
	NMS	8	Fixed	1.22 [1.18–1.27]	46	
	MS	4	Random	1.23 [1.03–1.49]	81	
	*Ethnicity*					
	Asian	9	Random	1.27 [1.18–1.35]	71	
	Non-Asian	4	Fixed	1.23 [1.12–1.37]	32	
	*Cut-off value*					
	<3.0	5	Fixed	1.27 [1.20–1.32]	11	
	≥3.0	8	Random	1.22 [1.12–1.33]	73	

HR: hazard ratio; CI: confidence interval; OS: overall survival; CSS: cancer-specific survival; DFS: disease-free survival; RFS: recurrence-free survival; GT: gastrointestinal tract; NMS: nonmetastatic stage; MS: metastatic stage.

**Table 3:** Meta-analysis of HRs from multivariate survival analysis.

Outcome	Groups	Number of studies	Model	HR (95% CI)	*I* ^2^ (%)	Egger test
OS	*All study*	17	Random	1.21 [1.31–1.38]	73	0.028
	*Tumor site*					
	Esophagus	2	Fixed	1.25 [1.13–1.29]	0	
	Stomach	3	Fixed	1.11 [1.03–1.19]	39	
	Colorectal	8	Fixed	1.25 [1.19–1.30]	36	
	Liver	2	Random	1.37 [1.10–1.69]	77	
	Pancreas	2	Random	1.67 [0.90–3.03]	95	
	*Anatomic structure*					
	GT	13	Fixed	1.22 [1.18–1.25]	46	
	Non-GT	4	Random	1.59 [1.23–2.04]	85	
	*Stage*					
	NMS	9	Random	1.33 [1.20–1.49]	78	
	Mixed	3	Random	1.12 [1.01–1.25]	57	
	MS	5	Fixed	1.32 [1.23–1.41]	27	
	*Ethnicity*					
	Asian	13	Random	1.27 [1.18–1.37]	78	
	Non-Asian	4	Fixed	1.35 [1.20–1.49]	0	
	*Cut-off value*					
	<3.0	7	Random	1.35 [1.19–1.33]	83	
	≥3.0	10	Fixed	1.25 [1.16–1.35]	63	
	*Treatment*					
	With surgery	13	Random	1.32 [1.28–1.37]	79	
	No surgery	4	Fixed	1.27 [1.18–1.35]	0	

CSS	*All study*	5	Random	1.18 [1.04–1.34]	65	0.105
	*Tumor site*					
	Colorectal	2	Fixed	1.47 [1.19–1.82]	0	
	*Anatomic structure*					
	GT	4	Random	1.23 [1.02–1.52]	72	
	*Stage*					
	Mixed	2	Fixed	1.06 [0.99–1.22]	26	
	MS	2	Fixed	1.47 [1.19–1.82]	0	
	*Ethnicity*					
	Asian	3	Random	1.20 [0.96–1.52]	77	
	Non-Asian	2	Random	1.19 [0.95–1.49]	12	
	*Cut-off value*					
	<3.0	2	Fixed	1.12 [1.04–1.22]	44	
	≥3.0	3	Random	1.27 [0.93–1.69]	79	

DFS/RFS	*All study*	12	Random	1.26 [1.16–1.38]	83	0.025
	*Tumor site*					
	Stomach	2	Fixed	1.06 [0.98–1.18]	25	
	Colorectal	6	Random	1.16 [1.09–1.25]	52	
	Liver	2	Fixed	1.37 [1.25–1.49]	16	
	*Anatomic structure*					
	GT	9	Fixed	1.12 [1.09–1.16]	46	
	Non-GT	3	Random	1.59 [1.19–2.08]	89	
	*Stage*					
	NMS	8	Random	1.30 [1.15–1.47]	85	
	MS	3	Random	1.30 [1.08–1.56]	85	
	*Ethnicity*					
	Asian	8	Random	1.25 [1.11–1.41]	88	
	Non-Asian	4	Random	1.32 [1.10–1.59]	32	
	*Cut-off value*					
	<3.0	5	Random	1.45 [1.14–1.89]	90	
	≥3.0	7	Random	1.18 [1.09–1.28]	74	

HR: hazard ratio; CI: confidence interval; OS: overall survival; CSS: cancer-specific survival; DFS: disease-free survival; RFS: recurrence-free survival; GT: gastrointestinal tract; NMS: nonmetastatic stage; MS: metastatic stage.

**Table 4:** *P* values of meta-regressions.

Covariate	Meta-analysis of MVA HRs	
	OS	DFS/RFS
*Anatomic structure*	0.015	0.043
*Tumor site*		
Esophagus	0.118	0.003
Stomach	0.016	0.015
Colorectal	0.094	0.018
Liver	0.752	0.002
Pancreas	DR	DR
*Tumor stage*		
NMS	0.983	0.277
Mix	0.097	DR
MS	DR	0.316
*Ethnicity*	0.328	0.637
*Cut-off value*	0.120	0.160
*Beyond cut-off*	0.981	0.720
*Treatment*	0.772	0.579

MVA: multivariate analysis; HR: hazard ratio; OS: overall survival; DFS: disease-free survival; RFS: recurrence-free survival; NMS: nonmetastatic stage; MS: metastatic stage; DR: dropped because of collinearity.
