# Supplementary material for: Pretreatment Lymphocyte Monocyte Ratio Predicts Long-Term Outcomes in Patients with Digestive System Tumor: A Meta-Analysis
Source: Gastroenterol Res Pract. 2016 Aug 9;2016:9801063. doi: 10.1155/2016/9801063 (PMC4993921; doi:10.1155/2016/9801063)
Supplement: Supplementary file 1 — Example of search criteria in “PubMed”: The following keywords were used in various forms and combinations for “Title/Abstract” based search: “cancer”, “tumor”, “carcinoma”, “neoplasm”, “adenocarcinoma”, “malignant”, “oncology”, “lymphocyte monocyte ratio”, and “monocyte lymphocyte ratio”. Additionally, “neoplasm” was also used in “Medical Subject Headings” based search. Table S1：This scale is an eight-item instrument that allows for assessment of patient population and selection, study comparability, follow-up, and outcome of interest. Interpretation of the scale is performed by awarding stars, for high-quality elements. Stars are then added up and use to compare study quality in a quantitative manner. A study can be awarded a maximum of one star for each numbered item within the Selection and Outcome categories. A maximum of two stars can be given for Comparability. Quoted phrases are provided in the scale to allow for adjustment to particular studies. Table S2：The scale includes eight items in total with four items in selection category, one item in comparability category, and three items in outcome category. Stars were given to high-quality elements. Having seven or more stars is considered good quality. Table S3: Summary table of studies included in meta-analysis. All studies were conducted between 2013-2016. Country described where the study conducted. Tumor site included colorectal, stomach, esophageal, and liver and pancreas. Treatment described whether the patients received surgical or not. Tumor stage was most often described according to distant metastasis status. For this table, studies were grouped as non-metastatic stage (NMS), metastatic stage (MS) or mixed stage. Clinical outcomes include overall survival (OS), cancer-specific survival (CSS), relapse-free survival (RFS), and disease-free survival (DFS). [file 9801063.f1.pdf]

Supplementary material

Example of search criteria in "PubMed":

((("Neoplasms"[Mesh]) OR (((((((neoplasm[Title/Abstract]) OR cancer[Title/Abstract]) OR carcinoma[Title/Abstract]) OR tumor[Title/Abstract]) OR adenocarcinoma[Title/Abstract]) OR oncology[Title/Abstract]) OR malignant[Title/Abstract]))) AND (((((LMR[Title/Abstract]) OR lymphocyte monocyte ratio[Title/Abstract]) OR lymphocyte-monocyte ratio[Title/Abstract]) OR lymphocyte/monocyte ratio[Title/Abstract]) OR lymphocyte to monocyte ratio[Title/Abstract])

Supplementary Table S1

Table S1. Newcastle-Ottawa scale used for methodological quality assessment of Cohort Study

**Check List**
**Selection**
(1) Representativeness of the exposed cohort
(a) Truly representative of the average 'digestive system cancer patients' in the community (1 star)
(b) Somewhat representative of the average 'digestive system cancer patients' in the community (1 star)
(c) Selected group of users (e.g. nurses, volunteers)
(d) No description of the derivation of the cohort
(2) Selection of the non-exposed cohort
(a) Drawn from the same community as the exposed cohort (1 star)
(b) Drawn from a different source
(c) No description of the derivation of the non-exposed cohort
(3) Ascertainment of exposure (Proof of digestive system caner and LMR measurement)
(a) Secure record (eg. Surgical records) (1 star)
(b) Structured interview (1 star)
(c) Written self-report
(d) No description
(4) Demonstration that outcome of interest was not present at start of study
(a) Yes (1 star)
(b) No
**Comparability**
(1) Comparability of cohorts on the basis of the design or analysis
(a) Study controls for '1, 2, 3' (one star was assigned if more than 1 of these 3 characteristics were reported; 0 star was assigned if none of these 3 characteristics was reported)
(b) Study controls for '4, 5, 6, 7, 8, 9' (one star was assigned if more than 1 of these 6 characteristics was reported; 0 star was assigned if none of these 6 characteristics was reported)

Underlined and quoted phrases are provided in the scale to allow for adjustment to particular studies.

Comparability variables: 1, Infection; 2, Immune system disorders; 3, Anti-inflammation drugs taken; 4, Age; 5, Gender; 6, Tumor Stage; 7, Metastasis; 8, Tumor grade; 9, Other

Table S1. Newcastle-Ottawa scale used for methodological quality assessment of Cohort Study (continued)

**Check List**
**Outcome**
(1) Assessment of outcome (Death or recurrence)
(a) Independent blind assessment (1 star)
(b) Record linkage (1 star)
(c) Self-report
(d) No description
(2) Was follow-up long enough for outcomes to occur? (Death or recurrence)
(a) Yes ('2-5 years') (1 star)
(b) No
(3) Adequacy of follow-up of cohorts
(a) Complete follow-up: all subjects accounted for (1 star)
(b) Subjects lost to follow-up unlikely to introduce bias: small number lost '25%' or description provided of those lost (1 star)
(c) Follow-up rate '75%' and no description of those lost
(d) No statement

Supplementary Results

Table S2. Assessment of Quality of Studies

Author	Year	Selection				Comparability	Outcome assessment			Score
		1	2	3	4	5	6	7	8	
Chan	2016	*	*	*	*	*	*	*	*	********
Facciorusso	2016	*	*	*	*	*	*	*	*	********
Hsu	2016	*	*	*	*	*	*	*	-	*******
Li[1]	2016	*	*	*	*	**	*	*	*	*********
Li[2]	2016	*	*	*	*	**	*	*	*	*********
Wu	2016	*	*	*	*	**	*	*	*	*********
Deng	2015	*	*	*	*	**	*	*	*	*********
Han	2015	*	*	*	*	**	*	*	*	*********
Huang	2015	*	*	*	*	*	*	*	-	*******
Kozak	2015	*	*	*	*	-	*	*	*	********
Lin	2015	*	*	*	*	**	*	*	*	*********
Liu	2015	*	*	*	*	**	*	*	-	********
Neal	2015	*	*	*	*	-	*	*	*	*******
Neofytou	2015	*	*	*	*	*	*	*	*	********
Ozawa	2015	*	*	*	*	*	*	*	-	*******
Qi	2015	*	*	*	*	*	*	*	-	*******
Shibutani	2015	*	*	*	*	*	*	*	*	********
Song	2015	*	*	*	*	*	*	*	-	*******
Xiao	2015	*	*	*	*	*	*	*	*	********
Stotz[1]	2014	*	*	*	*	*	*	*	*	********
Stotz[2]	2014	*	*	*	*	*	*	*	*	********
Zhou	2014	*	*	*	*	**	*	*	*	*********

-: zero point, *: One point, **: Two points

Table S3. Detailed Characteristics of included studies

Study	Year	No.	Country	Male (%)	Age	Site	Therapy	Stage	Follow up	Cut-off	Beyond cut-off (%)	Outcome	MVA	Method to get UVA HR
Chan	2016	1623	Australia.	801 (49.4)	NA	Colorectal	with-Surg	NMS	52.0[md]	2.38	797 (49.1)	OS	YES	Reported
Facciorusso	2016	127	Italy	83 (69.2)	66.0[md]	Colorectal	no-Surg	MS	60.0[md]	3.96	70 (55.1)	OS, RFS	YES	Reported
Hsu	2016	926	China	583 (63.0)	NA	Stomach	with-Surg	Mixed	32.0[md]	4.8	416 (44.9)	OS	YES	Estimated
Li[1]	2016	144	China	77 (53.5)	62[me]	Pancreas	with-Surg	NMS	14[md]	2.8	68 (47.2)	OS, RFS	YES	Estimated
Li[2]	2016	5336	China	3167 (59.4)	59[md]	Colorectal	with-Surg	NMS	55.2[md]	2.83	3988 (74.7)	OS, DFS	YES	Reported
Wu	2016	450	China	391 (86.9)	49.6[me]	Liver	with-Surg	MS	45.5[md]	3.77	269 (59.8)	OS, RFS	YES	Reported
Deng	2015	389	China	282 (72.5)	65.0[md]	Stomach	with-Surg	Mixed	24.0[md]	4.95	148 (38.0)	OS,CSS,DFS	YES	Estimated
Han	2015	218	China	177 (81.2)	60.5[md]	Esophagus	with-Surg	NMS	38.6[md]	2.57	173 (79.4)	OS, DFS	YES	Reported
Huang	2015	348	China	303 (87.1)	59.2[me]	Esophagus	with-Surg	NMS	NA	2.93	145 (41.7)	CSS	YES	Reported
Kozak	2015	129	USA	55 (42.6)	67.0[md]	Colorectal	with-Surg	NMS	24.7[md]	2.6	64 (49.6)	OS, DFS	YES	Estimated
Lin	2015	210	China	185 (88.1)	NA	Liver	with-Surg	NMS	34.8[md]	3.23	144 (68.6)	OS, RFS	YES	Reported
Liu	2015	326	China	283 (86.8)	59.2[me]	Esophagus	with-Surg	NMS	49[md]	2.30	158 (48.5)	OS	YES	Reported
Neal	2015	302	UK	192 (63.6)	66.0[md]	Colorectal	with-Surg	MS	29.7[md]	2.35	219 (72.5)	OS, CSS	NO	Reported
Neofytou	2015	140	UK	88 (62.9)	NA	Colorectal	with-Surg	MS	33.0[md]	3.0	65 (46.4)	OS,CSS, DFS	YES	Reported
Ozawa	2015	117	Japan	69 (58.9)	62[me]	Colorectal	with-Surg	MS	39.0[md]	3.0	79 (68.0)	CSS, DFS	YES	Reported

Abbreviation: [md] Median; [me] Mean; Surg, surgery; NMS, non-metastatic stage; MS, metastatic stage; NA, not available; OS, overall survival; DFS, disease-free survival; RFS, recurrence-free survival; CSS, cancer-specific survival; MVA, multivariate analysis; UVA, univariate analysis; HR, hazard ratio

Table S3. Detailed Characteristics of included studies (continued)

Study	Year	No.	Country	Male (%)	Age	Site	Therapy	Stage	Follow up	Cut-off	Beyond cut-off (%)	Outcome	MVA	Method to get UVA HR
Qi	2015	211	China	134 (63.5)	61.2^{me}	Pancreas	no-Surg	Mixed	NA	3.3	109 (51.7)	OS	YES	Estimated
Shibutani	2015	104	Japan	59 (56.7)	64.0^{md}	Colorectal	no-Surg	MS	22.4^{md}	3.38	66 (63.5)	OS	YES	Reported
Song	2015	177	Korea	83 (46.9)	52.0^{md}	Colorectal	no-Surg	MS	3.1^{md}	3.4	64 (36.1)	OS	YES	Estimated
Xiao	2015	280	China	175 (62.5)	NA	Rectum	with-Surg	NMS	52.0^{md}	3.78	140 (50.0)	DFS	YES	Reported
Stotz[1]	2014	372	Austria	217 (58.3)	64.0^{md}	Colon	with-Surg	NMS	68.0^{md}	2.83	105 (28.2)	OS, RFS	YES	Reported
Stotz[2]	2014	474	Austria	256 (54.0)	64.6^{me}	Pancreas	with-Surg	Mixed	36.0^{md}	2.8	201 (42.4)	CSS	YES	Reported
Zhou	2014	426	China	304 (71.4)	NA	Stomach	with-Surg	NMS	39.6^{md}	4.32	242 (56.8)	OS, RFS	YES	Reported

Abbreviation: ^{md} Median; ^{me} Mean; Surg, surgery; NMS, non-metastatic stage; MS, metastatic stage; NA, not available; OS, overall survival; DFS, disease-free survival; RFS, recurrence-free survival; CSS, cancer-specific survival; MVA, multivariate analysis; UVA, univariate analysis; HR, hazard ratio.
